# Recent Salmon Declines: A Result of Lost Feeding Opportunities Due to Bad Timing?

**DOI:** 10.1371/journal.pone.0012423

**Published:** 2010-08-27

**Authors:** Cedar M. Chittenden, Jenny L. A. Jensen, David Ewart, Shannon Anderson, Shannon Balfry, Elan Downey, Alexandra Eaves, Sonja Saksida, Brian Smith, Stephen Vincent, David Welch, R. Scott McKinley

**Affiliations:** 1 Department of Arctic and Marine Biology, University of Tromsø, Tromsø, Norway; 2 Fisheries and Oceans Canada, Campbell River, British Columbia, Canada; 3 Centre for Aquaculture and Environmental Research, The University of British Columbia and Department of Fisheries and Oceans, West Vancouver, British Columbia, Canada; 4 British Columbia Centre for Aquatic Health Sciences, Campbell River, British Columbia, Canada; 5 Seymour Salmonid Society, North Vancouver, British Columbia, Canada; 6 Kintama Research Corp, Nanaimo, British Columbia, Canada; National Oceanic and Atmospheric Administration/National Marine Fisheries Service/Southwest Fisheries Science Center, United States of America

## Abstract

As the timing of spring productivity blooms in near-shore areas advances due to warming trends in global climate, the selection pressures on out-migrating salmon smolts are shifting. Species and stocks that leave natal streams earlier may be favoured over later-migrating fish. The low post-release survival of hatchery fish during recent years may be in part due to static release times that do not take the timing of plankton blooms into account. This study examined the effects of release time on the migratory behaviour and survival of wild and hatchery-reared coho salmon (*Oncorhynchus kisutch*) using acoustic and coded-wire telemetry. Plankton monitoring and near-shore seining were also conducted to determine which habitat and food sources were favoured. Acoustic tags (n = 140) and coded-wire tags (n = 266,692) were implanted into coho salmon smolts at the Seymour and Quinsam Rivers, in British Columbia, Canada. Differences between wild and hatchery fish, and early and late releases were examined during the entire lifecycle. Physiological sampling was also carried out on 30 fish from each release group. The smolt-to-adult survival of coho salmon released during periods of high marine productivity was 1.5- to 3-fold greater than those released both before and after, and the fish's degree of smoltification affected their downstream migration time and duration of stay in the estuary. Therefore, hatchery managers should consider having smolts fully developed and ready for release during the peak of the near-shore plankton blooms. Monitoring chlorophyll *a* levels and water temperature early in the spring could provide a forecast of the timing of these blooms, giving hatcheries time to adjust their release schedule.

## Introduction

Increasing surface temperatures in western North America during the last half-century [1] have led to earlier spring freshets [2], [3] and advancing plankton blooms in near-shore and open ocean areas [4]–[6]. This trend towards an earlier productivity bloom may favour earlier out-migrating smolts and species [3], as smolts migrating out to marine feeding areas at the time of the plankton blooms may be more likely to reach a critical body size before summer, allowing them to survive the winter [4], [7], [8]. In fact, the peak out-migration period for some populations of wild juvenile Pacific salmon (*Oncorhynchus* spp.) has already shown signs of advancement [8], [9], which may be a simple reflection of warmer temperatures causing earlier development, a selective pressure favouring earlier-migrating fish, or both.

The “match/mismatch” concept states that the success of marine fish larvae in temperate regions depends on the timing of hatching relative to the productivity blooms [10]. A match may lead to greater survival, due to higher food availability at a critical time in the fish's life cycle. Similarly, the transition from fresh to marine waters is a stressful time in the life history of anadromous fishes such as salmonids. Thus, matching the timing of the smolt migration with the zooplankton bloom may increase the population's overall survival.

**Figure 1:**
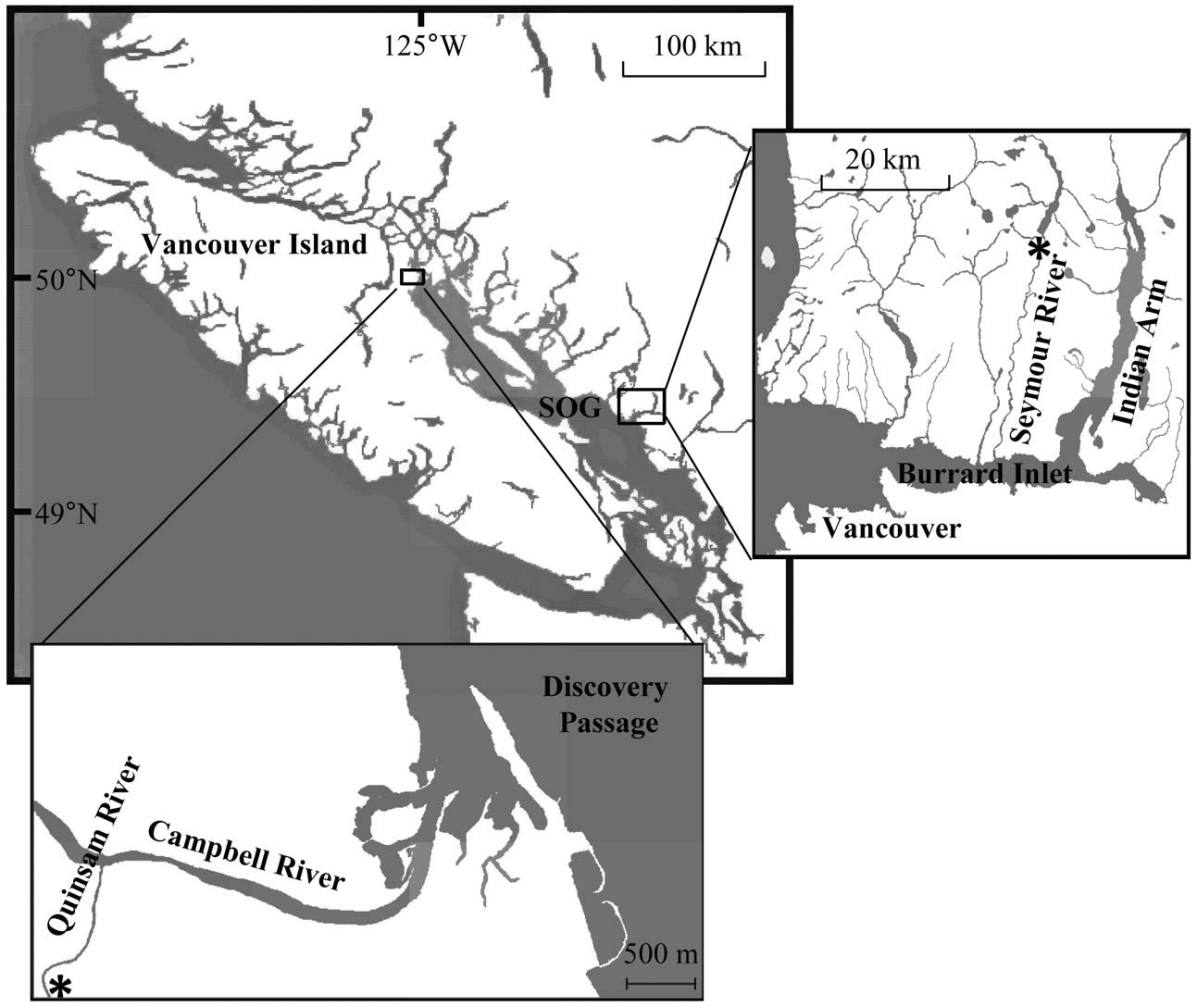
The study area, including the Seymour and Quinsam Rivers, Discovery Passage and the Strait of Georgia (SOG), Indian Arm and Burrard Inlet. Release locations are denoted by an asterix (*).

Salmon enhancement programs in the US and Canada have been releasing hatchery-reared salmon during the last half-century to increase population sizes. However, since the mid-90s, smolt-to-adult survival rates for hatchery coho salmon (coho; *O. kisutch*) entering the Strait of Georgia (Fig. 1) have been on average 0.9% (SD±0.1%), which is down from 8.8% (±1.6%) survival during the 1980s [8]. The cause of this decline in marine survival is unknown, though it has been suggested that long-term genetic effects and physiological deficiencies may contribute [11].

Most salmon hatcheries run by Fisheries and Oceans Canada time the release of enhanced coho smolts according to guidelines established in the early 1980s [12]. These procedures are based on observations that the survival rates for coho were maximized when the smolts were released near the third week of May at a size of 20–25 g [12]. However, recent changes in the magnitude and timing of marine productivity in the Strait of Georgia [13] have likely resulted in a mismatch between the timing of the smolts' releases and the occurrence of the spring plankton blooms they rely on as a primary food source. Thus, as wild fish are migrating to ocean feeding grounds in time with the spring freshet and other environmental cues (e.g. temperature and light [14]–[17]), and hatchery fish continue to have static release times, the low post-release survival of hatchery coho may simply be a case of bad timing [8].

We hypothesized that the overall survival of hatchery-reared coho salmon smolts would increase if their release coincided with a plankton bloom. This hypothesis was tested by correlating local plankton productivity at the time of the smolt release with the smolt-to-adult survival of hatchery-reared coho salmon—i.e. return rates were compared between groups of coded-wire tagged smolts released during varied levels of marine productivity. Detailed smolt movement data in the riverine, estuarine and near-shore areas were acquired through population surveys (by seine sampling) and acoustic telemetry. Physiological comparisons between release groups, and analyses of stomach contents and size effects on survival added further background to the telemetry results.

## Methods

All work involving live fish reported in this paper was reviewed and pre-approved as meeting or exceeding the standards laid out by the Canadian Council on Animal Care. The project guidelines were specifically approved by The University of British Columbia's Committee on Animal Care, Suite 102, 6190 Agronomy Road, Vancouver, British Columbia (permit A06-0153).

Data from three telemetry projects are examined in this study. The fine-scale behaviour of out-migrating wild and hatchery-reared coho smolts was monitored using acoustic telemetry and seine-netting in the Quinsam and Campbell Rivers (Fig. 1). Early and late releases of hatchery coho from the Seymour River (Fig. 1) were also compared using acoustic telemetry. Finally, the smolt-to-adult survival of coho in relation to release timing was assessed through coded-wire tag (CWT) data from the Quinsam River.

### Plankton Surveys

Discovery Passage (Fig. 1), at the northern tip of the Strait of Georgia, is a migratory route for many salmon stocks. Plankton surveys were conducted weekly in this marine area from 2007 to 2009 between February and July, to estimate the timing of spring plankton blooms.

Phytoplankton and chlorophyll *a* were sampled at a 5 m depth with a LaMotte water sampler (Dynamic Aqua-Supply, Vancouver, British Columbia). Each 1 L water sample was filtered using a syringe and filter system. The filter paper was stored in the dark and taken to the lab for analysis of chlorophyll *a* and phaeopigments (products of chlorophyll degradation found within algal cells). Approximately 125 ml of the water sample was put into a 150 ml sample container with 10–12 drops of Lugol's solution added to preserve the sample. In addition to the discrete sampling, a 50 µm conical phytoplankton net was used to collect a vertical tow from 5 m to the surface. The net was rinsed and poured into a 125 ml container with 10–12 drops of Lugol's solution. The proportions of the different plankton groups in a 1 ml sub-sample of the discrete water sample were estimated using a Sedgwick-Rafter cell. Another 1 ml subsample was taken from the 125 ml vertical tow sample and the analysis was repeated.

Zooplankton sampling was performed during the same time period, with three replicates obtained on each trip. A 0.5 m×2.0 m, 250 µm conical plankton net mounted on a fixed metal frame with a removable, weighted cod-end sample container was lowered to a depth of 20 m (near bottom) and pulled up at a steady 1 m·s^−1^ rate to the surface. A hydraulic winch allowed for consistent retrieving of the net, and slight maneuvering of the boat kept it vertical. The net was rinsed in seawater to concentrate the plankton in the cod-end. The three samples were collected in labeled 250 ml containers and preserved with 20 ml of 37% formalin.

In the laboratory, the preserved plankton net samples were poured into three 250 ml graduated cylinders and allowed to settle for 1–2 hours. The total biomass of plankton was estimated using the settled volume, reported as the height in millimeters from the cylinder bottom. The volume was then recorded as millimeters of plankton per cubic meter of seawater filtered through the net. A subsample was prepared by splitting the original sample using a Folsom Plankton Splitter (Aquatic Research Instruments, Idaho) and counted using a plankton counter. The zooplankters were identified to order with the aid of a binocular microscope.

### Fish Surveys

Juvenile salmonids were sampled weekly or bi-weekly by beach seine at multiple sites in the Campbell River estuary and Discovery Passage near-shore from late April to late June 2007, 2008 and 2009. A 13.5m long×2.9 m deep seine net consisting of three sections (two outer 4.5 m wings of 1 cm stretch mesh and a 4.6 m center bunt section of 0.6 cm stretch mesh) was set using a jet boat. Bridles were attached to the net gable end system with ropes marked off at 100 m. The net was pulled off the boat by a crew on the beach, secured to a tow pole on the boat and set in a horseshoe shape to sample an area of 100 m^2^. The net was pulled slowly to shore by both crews. All catch was enumerated and identified to species and origin (hatchery, wild or unknown). The beach seine was repeated if insufficient numbers of coho were captured on the first set. Nearly all hatchery-origin coho had their adipose fin clipped. A small percentage of hatchery-origin fish, with or without an adipose clip, were coded-wire tagged. All coho were weighed, measured and inspected for any physical abnormalities. Stomachs were excised and placed in 125 ml of 10% formalin. The stomach contents were identified and enumerated.

### Fish Telemetry

Acoustic telemetry methods were used to monitor wild and hatchery coho salmon smolts (n = 140) released at different times from two rivers that enter the Strait of Georgia (Fig. 1). Acoustic receivers (VR2, VEMCO Ltd, Nova Scotia) were deployed in the Campbell (8 km from release to estuary) and Seymour Rivers (20 km from release to estuary) to monitor smolt migration out to the ocean. Receiver arrays from the Pacific Ocean Shelf Tracking (POST) project recorded marine movements.

River-caught (“wild”; n = 40) and hatchery-reared (n = 40) coho smolts from the Quinsam River (entering the Campbell River) were tagged during 2006 to monitor smolt post-release survival and migratory behaviour [18] (Table 1). During 2007, 60 hatchery-reared coho smolts from the Seymour River were acoustically tagged with V7-2L tags (7·18.5 mm, VEMCO Ltd) following the same methods. The fish were released at the Seymour hatchery in two groups (Table 1) to study the effects of release timing on their freshwater and marine behaviour and survival. The release groups had similar masses (first: 23.3±1.9 g, second: 22.4±2.0 g) and fork lengths (first: 12.9±0.3 cm, second: 12.8±0.3 cm). Wild smolts were captured in baited minnow traps in the Seymour River for one month; however none were large enough to tag (4.1±0.3 g, 7.1±0.3 cm), so a comparison of wild and hatchery coho was not possible in the Seymour River.

**Table 1:** Acoustically tagged wild (W) and hatchery (H) coho salmon released at the Quinsam Hatchery (Rel 2006) and Seymour Hatchery (Rel 2007), including the number released (N Rel) and the number and proportion of smolts detected in the respective estuaries (N Est, % Est).

Brood	Rel	Rel Date	Group	N Rel	N Est	% Est
2004	2006	21/05/2006	H	20	17	85%
		21/05/2006	W	20	19	95%
		22/05/2006	H	20	15	75%
		22/05/2006	W	20	20	100%
2005	2007	25/04/2007	H	35	19	54%
		04/05/2007	H	25	10	40%

From 2006 to 2008, releases of coho from the Quinsam Hatchery were tagged with coded-wire tags (n = 266,692) according to methods described in [19]. Releases were from the hatchery during early April to late May (Table 2). The fish were all smolted in time for release, although no formal saltwater challenge trial was done. The release groups were raised to approximately the same mean size (25 g) to eliminate any size bias. During 2008, there were two size groups (25 g and 30 g) released during mid-May to test the influence of size at release on overall survival.

**Table 2:** Groups of coho salmon from the Quinsam Hatchery tagged with coded-wire tags during 2006–2008, including the number (N) and proportion of returning (Ret) jacks (J) and adults (A) for each release group (Rel).

Brood	Rel	J Ret	A Ret	Rel Date	N Rel	N J Ret	% J Ret	N A Ret	% A Ret
2004	2006	2006	2007	25/04/2006	21,849	24	0.11%	97	0.44%
				17/05/2006	21,828	38	0.17%	152	0.70%
				20/05/2006	21,857	43	0.20%	146	0.67%
				24/05/2006	21,812	41	0.19%	123	0.56%
2005	2007	2007	2008	25/04/2007	23,353	21	0.09%	79	0.34%
				09/05/2007	23,445	56	0.24%	218	0.93%
				16/05/2007	23,204	56	0.24%	175	0.75%
				23/05/2007	20,738	25	0.12%	97	0.47%
2006	2008	2008	2009	24/04/2008	22,032	10	0.05%	114	0.52%
				08/05/2008	22,125	35	0.16%	341	1.54%
				08/05/2008	22,732*	30	0.13%	414	1.82%
				22/05/2008	21,717	18	0.08%	285	1.31%

The asterix (*) denotes the 30 g body mass release group of 2008, whereas all the other release groups had a mean mass of approximately 25 g.

Physiological analyses were conducted on the coho populations used in the telemetry studies for a baseline to help understand the migration and survival results. The fish (n = 30 per group) were euthanized in a bath containing buffered tricaine methanesulphonate (200 ppm TMS; 400 ppm sodium bicarbonate). The hatchery fish were not fed for 24–48 hours prior to sampling. All fish were weighed and measured so that their condition factors (mass·fork length^−3^) could be calculated. The tails were severed using sterile blades and blood was collected to determine the number of differential leucocytes and erythrocytes [20]. Briefly, 5 µL of blood was placed into 995 µL of Hendricks's solution [21] and the number of erythrocytes was determined, using manual hemacytometer counts. A drop of blood was then placed onto a clean glass slide, smeared and stained with a modified Wright-Giemsa stain according to manufacturer's instructions. Twenty-five fields were systematically examined from each slide (one slide per fish) under oil immersion (1,000×magnification). The number of lymphocytes, neutrophils, thrombocytes, monocytes, and erythrocytes were recorded and the ratio of leucocytes to erythrocytes was calculated. The number of circulating leucocytes·mm^−3^ was determined by multiplying the above ratio with the number of erythrocytes determined from the hemacytometer counts.

The left gill arch from each fish was removed, placed in foil and frozen in liquid nitrogen. The gill samples were transported to the lab and stored at −80°C to be analysed for Na+/K+-ATPase enzyme activity [22]. After removal of the gills, the fish were dissected to determine the necropsy-based health assessment score (modified from [23]). The necropsy score was based on a total score of 22, which included ranking the appearance of external (skin, eyes, gills, fins, pseudobranch) and internal organs (amount of visceral fat, kidney, liver, spleen, gall bladder, posterior intestine). A score of 0 represented a normal appearing fish with no visceral fat stores, lesions, swellings, hemorrhage, discolorations, or any other signs of abnormalities. The liver was weighed and the hepatosomatic index was calculated as the ratio of liver mass to body mass. Kidney samples were taken and tested for *Renibacterium salmoninarum*, the causative agent of bacterial kidney disease (BKD), using an enzyme-linked immunosorbent assay (ELISA).

### Analyses

Regression analyses were done to examine correlations between chlorophyll *a* and phytoplankton levels. Physiological comparisons (e.g. stomach contents, Na+/K+-ATPase activity, blood cell counts) between wild, hatchery and release groups were conducted using the Mann-Whitney U test. Range-testing trials determined that acoustic receivers in both the Campbell River and Seymour River estuaries had near-100% detection efficiency. Thus, smolt survival to the river mouth was calculated as the percentage of fish to be detected by estuary receivers. The overall smolt-to-adult survival (the percentage of tagged fish that returned to the hatchery) was compared between release groups using the Z test. Significance was established at P<0.05.

## Results

### Plankton Surveys

**Figure 2:**
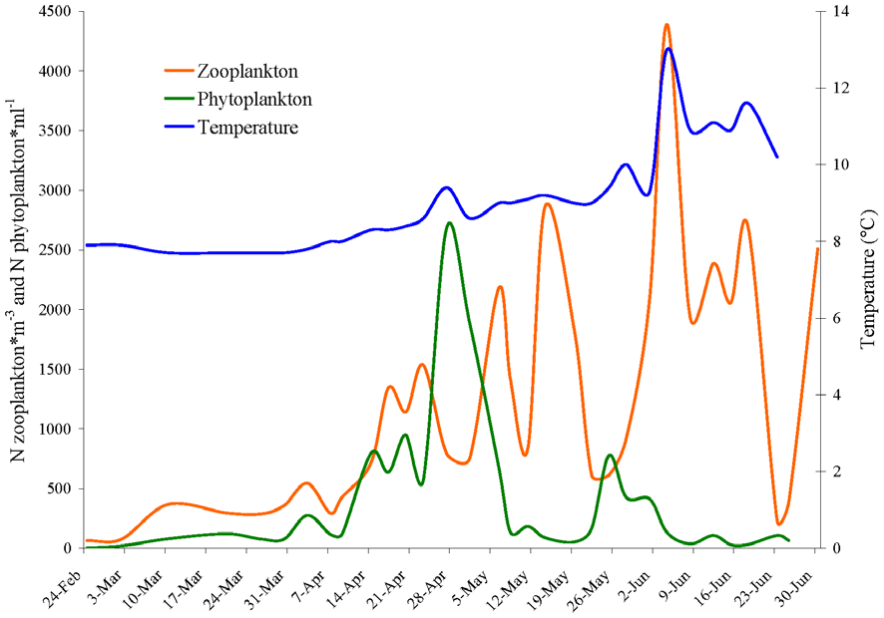
Zooplankton, phytoplankton and temperature in Discovery Passage during the 2009 monitoring period.

**Figure 3:**
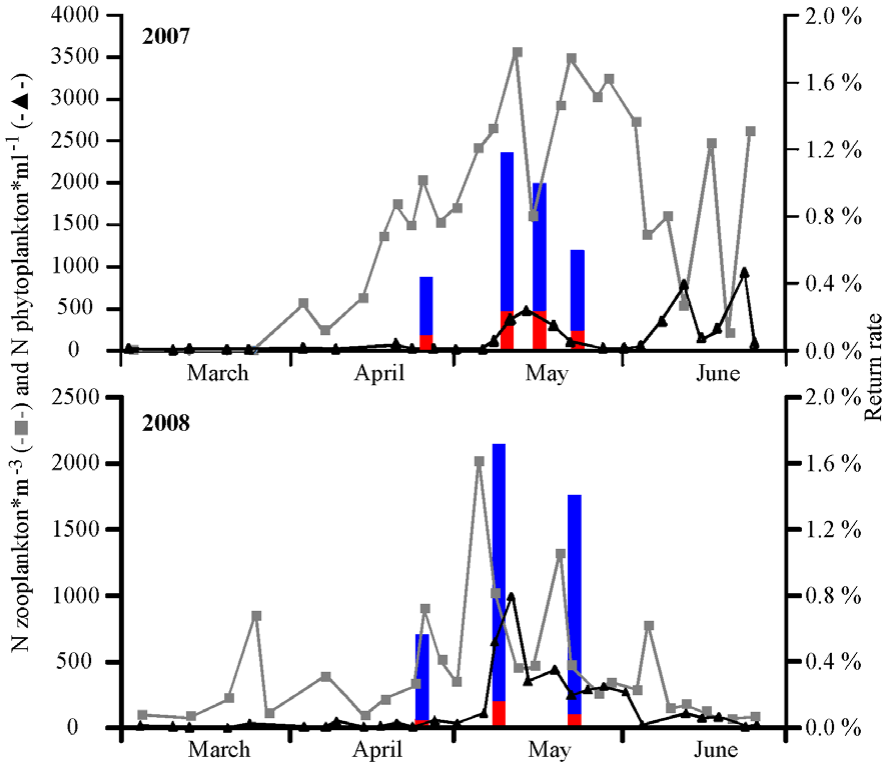
Zooplankton, phytoplankton, and return rates of coho salmon jacks (red bars) and adults (blue bars) released during 2007 and 2008.

**Figure 4:**
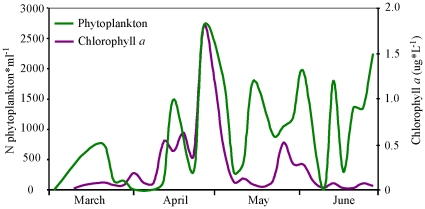
The relationship between chlorophyll *a* and phytoplankton abundance during 2009.

The phytoplankton bloom occurred earlier in 2009 (mid-April to early May) than in 2007 or 2008 (Fig. 2 and 3). Water temperatures reflected similar patterns. Chlorophyll *a* levels were positively correlated with phytoplankton titres (r = 0.65, P<0.01; Fig. 4) and an increase in phytoplankton production was followed by an increase in zooplankton density (Fig. 2). Copepods and cirripedians represented the majority of the zooplankters in the water during the sampling period. A high number of euphasid larvae were also present during the early spring of 2008 and 2009.

### Fish Surveys

Both wild and hatchery coho were caught in the near-shore areas and estuary from late May until the end of June. During periods of high marine productivity (2007: >500 phytoplankton·ml^−1^ and >2,500 zooplankton·m^−3^; 2008 and 2009: >500 phytoplankton·ml^−1^ and >1,000 zooplankton·m^−3^), very few coho salmon were captured in the estuary. However, during periods of lower marine productivity, many coho were present in the estuary—and none were caught in the near-shore marine areas.

**Figure 5:**
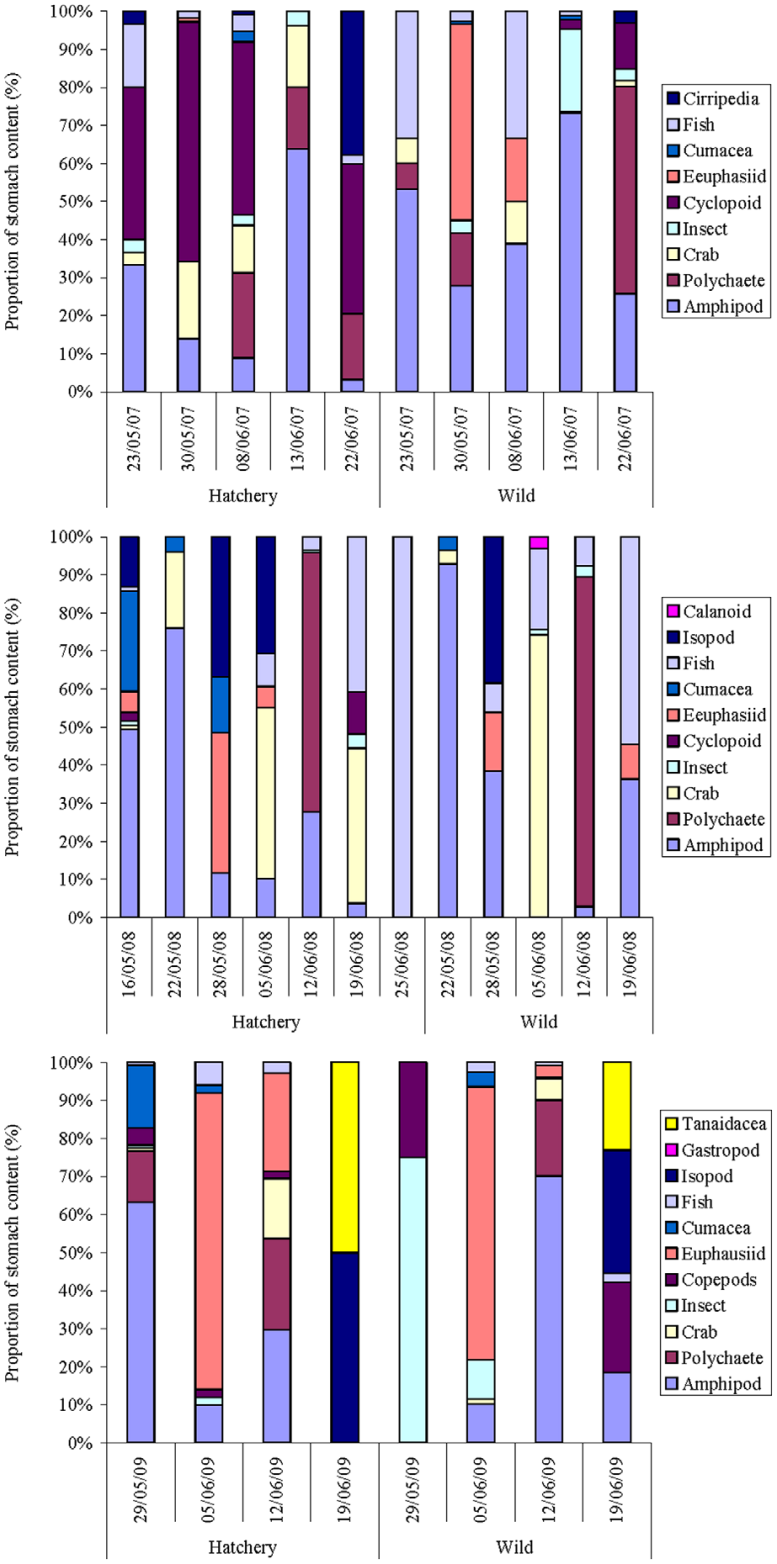
Stomach sample composition for wild and hatchery coho smolts in the Campbell River estuary during springs of 2007–2009.

**Figure 6:**
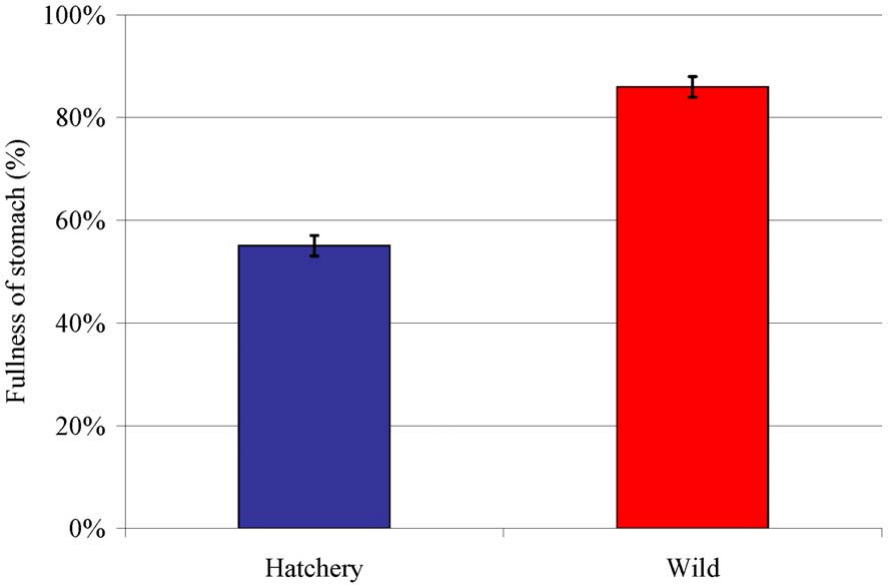
The mean stomach fullness of wild and hatchery coho salmon smolts in the Campbell River estuary and near-shore area from 2007 to 2009, with standard error.

From 2007 to 2009, the wild coho were more selective in their diet than the hatchery coho (Fig. 5). Of the prey species found in the stomachs contents, the major prey item made up 64% in the wild fish (mean, 2007–2009), whereas only 53% in hatchery fish (P = 0.02). The wild smolts (n = 343) also had fuller stomachs than the hatchery smolts (n = 222) from 2007–2009 (wild: 86%±30%, hatchery: 55%±30%; P<0.01; Fig. 6). Amphipods, euphasids and polycheates were the dominant groups observed in the stomach analysis; however, they were not a significant component of the zooplankton samples at the same time.

### Fish Telemetry

**Figure 7:**
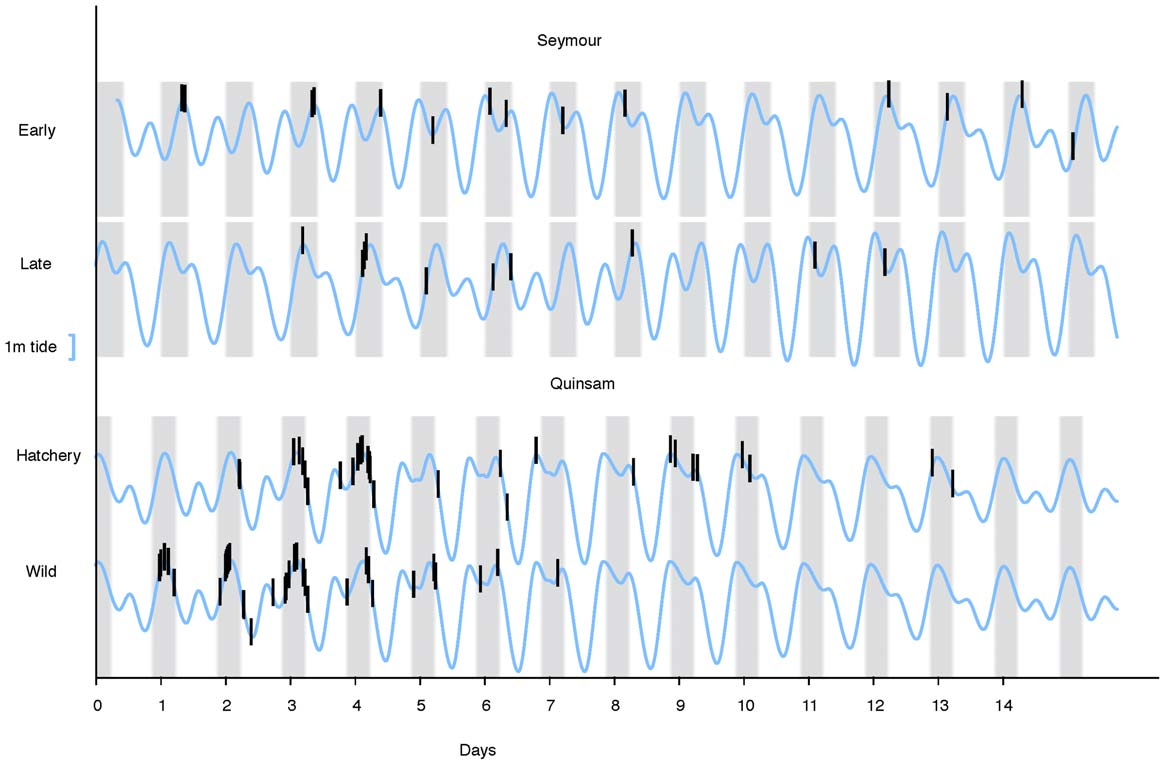
The first estuary detection of acoustically-tagged coho salmon (black bars) from the Quinsam (2006) and Seymour (2007) hatcheries. Tide height at the estuary is represented as a blue line and hours of darkness are represented by vertical gray bars. The x-origin for the two groups of Seymour coho is their release date, whereas that for the two groups of Quinsam fish is the midnight prior to the four separate release times (20 May 2006; individual release times not shown as each included both wild and hatchery fish). Not shown are five hatchery fish (three from the Seymour and two from the Quinsam) that arrived at the estuary more than two weeks after release.

During the 2006 acoustic tagging of wild and hatchery coho in the Quinsam River, the wild coho arrived at the estuary earlier, spent less time in the estuary and entered the marine environment earlier than hatchery smolts released at the same time [18] (Fig. 7). When hatchery smolts were released at different times from the Seymour hatchery in 2007, the first release group took longer to initiate migration (n = 24; 7±5 d, range 2–16 d) than the second group (n = 19; 1±1 d, range 0–7 d; P<0.03). The first group also took longer to reach the estuary from release (n = 19; 12±9 d, range 2–40 d) than the second group (n = 10; 7±3 d, range 4–13 d; P<0.05). However, both groups took approximately 3 days to complete the 20 km journey from the first receiver to the river mouth. After aggregating in the estuary for approximately one week, both release groups entered the marine environment at the same time (mean: 14 May 2007).

Nearly all of the smolts released from the Quinsam (92%) and Seymour (100%) hatcheries travelled downstream and arrived at the estuary during darkness, at high tide (Fig. 7) Whereas the Quinsam hatchery smolts had a freshwater survival of 80%, only 47% of the smolts migrating downstream in the longer Seymour River were detected in the estuary (Table 2). Smolts that were detected only briefly in the estuary and then moved quickly into the marine environment seemed to have a greater number of detections on marine receivers, although the sample size was small.

Of the 28 Seymour coho that were detected in the estuary, five were later recorded in the ocean. Two fish were detected in Indian Arm (Fig. 1) from 14 to 28 May, one of which was later recorded in the Strait of Georgia 3 June. Three were detected in Burrard Inlet from 5 to 25 May and presumably entered the Strait of Georgia. This number is very much a lower limit, due to a disruption of the receiver line during the season. Of the 80 Quinsam coho tagged acoustically, 43% of the wild and 32% of the hatchery fish were detected by ocean receivers [18]. As coho entering the Strait of Georgia generally residualise there, at least until the autumn [24], it was not possible to estimate marine survival using only acoustic tags.

The return rates of adults and early-maturing males (“jacks”) released during the zooplankton bloom were higher than those groups released either earlier or later. The smolt-to-jack/adult survival of CWT-tagged coho from the Quinsam hatchery was 1.5- to 3-fold greater when released within one week of high plankton productivity in Discovery Passage during 2007 (>500 phytoplankton·ml^−1^ and >2,500 zooplankton·m^−3^) and 2008 (>500 phytoplankton·ml^−1^ and >1,000 zooplankton·m^−3^; Fig. 3). During 2007, the second release group had the highest survival (Table 2), which was significantly greater than the first release group (Z = 8.0) and the third release group (Z = 2.1). The third group, in turn, had higher survival than the fourth release group (Z = 3.7). During 2008, the 30 g release group had higher smolt-to-adult survival than the 25 g group released on the same day (Z = 2.3), which, in turn, out-survived both those released before (Z = 10.6) and after (Z = 2.0). Smolt-to-jack survival rates followed the same trends; however there was no difference in jack survival between the 25 g and 30 g groups released on the same day.

The gill Na+/K+-ATPase activity was significantly higher in the wild than the hatchery smolts from the Quinsam River [18], and in the second than the first Seymour release group (Table 3). This second release group also had higher red blood cell counts, and lower white blood cell counts than the early group. The ELISA results for Bacterial Kidney Disease indicate that the Quinsam hatchery coho had low levels of BKD but were well below the DFO cutoff for release (an optical density less than 0.14).

**Table 3:** Health parameters of early and late release coho from the Seymour River Hatchery during April 2007, expressed as group mean (SD).

Health Parameter	Early Release	Late Release	Stats
Mass (g)	19.7 (0.92)	16.0 (0.4)	E = L
Fork Length (cm)	12.1 (0.2)	11.4 (0.1)	E = L
Condition Factor (M·FL^−3^)	1.10 (0.01)	1.07 (0.01)	E = L
Hepatosomatic Index (%)	0.80 (0.02)	0.80 (0.03)	E = L
Necropsy Score	4.8 (0.2)	4.3 (0.1)	E = L
Gill Na^+^/K^+^-ATPase Activity (µM ADP·mg protein^−1^·h^−1^)	6.3 (0.5)	8.2 (0.4)	E<L (P<0.01)
Total Number of Leukocytes (·10^4^·ml^−1^)	2.15 (0.19)	0.99 (0.15)	E>L (P<0.01)
Number of Lymphocytes (·10^4^·ml^−1^)	1.07 (0.11)	0.58 (0.11)	E>L (P<0.01)
Number of Thrombocytes (·10^4^·ml^−1^)	0.79 (0.11)	0.19 (0.06)	E>L (P<0.01)
Number of Neutrophils (·10^4^·ml^−1^)	0.29 (0.06)	0.22 (0.05)	E = L
Number of Monocytes (·10^4^·ml^−1^)	0.07 (0.07)	0.00 (0.00)	E = L
Number of Erythrocytes (·10^6^·ml^−1^)	1.02 (0.06)	1.43 (0.05)	E<L (P<0.01)

The results for the Quinsam smolts can be found in [18].

## Discussion

Coho salmon smolts released within a week of the near-shore marine productivity blooms had higher survival to adulthood than those released both before and after. Smolts were also less likely to be found in the estuary during periods of high marine productivity. Wild smolts were better foragers than hatchery smolts, and larger smolts had higher smolt-to-adult survival than those weighing 5 g less at release. The results of this study support the early-migrant [3] and critical-size [4], [7], [8] hypotheses.

Both wild and hatchery-reared coho smolts remained in the estuarine environment during periods of low marine productivity. When near-shore plankton levels were high, however, they left the estuary and entered the marine environment. Estuaries are typically risky for out-migrating salmon smolts due to high predator densities [25], [26]. Thus, if smolts remain in estuaries for an extended period of time before food cues draw them into marine waters, they may do so at a higher risk. This could be the reason that the acoustically-tagged smolts that passed quickly through the estuary were more likely to be detected again later on marine receivers. Furthermore, hatchery fish may be at a higher risk of predation, as they have been shown to have reduced predator-avoidance abilities [27], [28]. To increase the post-release survival rates of hatchery salmon, releases should occur during periods of high marine productivity so that smolts spend minimal time in high-risk areas.

Gill Na+/K+-ATPase activity levels were higher in the late (vs. early) Seymour release group and in the wild (vs. hatchery) Quinsam group, meaning that they were perhaps more smoltified and ready to enter saltwater [29]. The white blood cell (leukocyte) count data also support this conclusion. The late Seymour release group had lower leukocyte counts than the early group (not measured in the Quinsam groups), which is likely because cortisol—another indicator of the physical changes occurring during smoltification—is lympholytic (lymphocyte-killing) and immunosuppressive [30]. Thus, the smolts' state of physical readiness may explain why late Seymour and wild Quinsam smolts took less time to get to the estuary and spent less time in the estuary than the smolts from the other release groups. Reducing hatchery rearing densities may be used to accelerate smolt development when required, as this would reduce cortisol levels and increase levels of growth hormone [31].

Trawl surveys conducted in the Strait of Georgia during July and September 1997–2007 reported no difference between wild and hatchery coho in terms of the volume and composition of their stomach contents [31]. However, the seine surveys in the current study were done earlier (April to June), and found that wild coho had fuller stomachs and a more selective diet than the hatchery coho, possibly choosing higher quality foods. Hatchery juveniles, habituated to being fed pellets from the surface, may undergo a learning process after release in order to be able to recognize optimal prey types (possibly achieving wild-type feeding patterns by July). This time spent learning optimal foraging behaviour may add further risk to the early marine survival of the hatchery-reared smolts. If smolts must reach a certain mass by a certain time in order to survive the winter [4], [32], then losing time to learn how to feed may increase their mortality risk. Enhancement programs could start this foraging training early at the hatchery with live feed and/or underwater feeding systems to improve smolt post-release survival.

Benthic zooplankton, including amphipods, euphasids and polycheates, were dominant in the stomach content of coho smolts inhabiting the near-shore areas of Discovery Passage, but were not a significant component of the zooplankton samples taken in the water. Thus, the smolts may have been feeding selectively on those larger zooplankton species. However, it could be that the smaller prey had been digested more rapidly, leaving more of the larger animals behind. Another possibility is that the sampling was done during daylight hours only and many zooplankton species have diurnal vertical migrations, although the entire water column was sampled (from a depth of 20 m). The fact that the beach seining (stomach samples) was done within 15 m of shore and 3–5 m deep, whereas the plankton sampling was done further off-shore in over 20 m depth may have also contributed to the differences seen—i.e. the stomach samples may reflect the more epibenthic shore groceries. Adding an epibenthic sled sample to the beach seines would address this question in future studies.

There continues to be a lack of knowledge about the long-term trends and patterns of inter-annual variability in plankton population dynamics [33]. Developing regular monitoring programs to forecast the timing of annual plankton blooms could be a key to increasing hatchery survival rates. The ability to predict the blooms of favourite prey species for each species of salmon would be ideal. Hatchery programs in Alaska have historically used plankton abundance as a guide for timing the releases of pink (*O. gorbuscha*) and chum (*O. keta*) salmon [34]. However, discrete sampling at specific depths may not be the most efficient method to assess plankton production, as boat and crew time are expensive and laboratory analyses time consuming, making the quick turnaround of results difficult. From 2007–2009, chlorophyll *a* levels correlated positively to phytoplankton activity. Thus, chlorophyll *a* sampling would allow for the early detection of the small phytoplankton that precede the bloom of larger diatoms, which in turn precede the bloom of zooplankton. Along with accurate forecasts of water temperature, it should become possible to predict zooplankton blooms weeks in advance, giving hatchery managers enough flexibility to adjust feeding regimes so that smolts are adapted physically in time for their release. Multiple releases could be conducted over a protracted window of time as close as possible to the forecasted bloom, to reduce the risk of fish being underdeveloped or spending too much time in freshwater.

This study further demonstrates that environmental indices, such as water temperature and marine productivity, should be taken into account in the management of fish populations [35]. The success of enhancement programs and other mitigative strategies may depend on it. Similar experiments should follow in other marine areas, as local currents and conditions may differ. Species comparisons are recommended to determine whether earlier plankton blooms are affecting later migrants (e.g. coho and chinook salmon, *O. tshawytscha*) more than earlier migrants (e.g. pink and chum salmon). Long-term studies are also required to understand the relationship between climate patterns and marine productivity blooms. In addition to the long-term climate trends that may be advancing the spring plankton blooms, decadal regime shifts and El Niño events affect marine productivity on a shorter time scale [36]. Therefore, until more is known about the dynamic interactions between plankton populations, and long- and short-term climate patterns, timing smolt releases to match the annual productivity bloom will be challenging. Releasing multiple groups of hatchery smolts during the probable high productivity period would be an adequate interim solution.
